# Metal–insulator transition tuned by oxygen vacancy migration across TiO_2_/VO_2_ interface

**DOI:** 10.1038/s41598-020-75695-1

**Published:** 2020-10-29

**Authors:** Qiyang Lu, Changhee Sohn, Guoxiang Hu, Xiang Gao, Matthew F. Chisholm, Ilkka Kylänpää, Jaron T. Krogel, Paul R. C. Kent, Olle Heinonen, P. Ganesh, Ho Nyung Lee

**Affiliations:** 1 Materials Science and Technology Division, Oak Ridge National Laboratory, Oak Ridge, TN 37831 USA; 2 Center for Nanophase Materials Sciences, Oak Ridge National Laboratory, Oak Ridge, TN 37831 USA; 3 Computational Physics Laboratory, Tampere University, P.O. Box 692, 33014 Tampere, Finland; 4 Computational Science and Engineering Division, Oak Ridge National Laboratory, Oak Ridge, TN 37831 USA; 5 Materials Science Division, Argonne National Laboratory, Lemont, IL 60439 USA; 6 Present Address: School of Engineering, Westlake University, Hangzhou, 310024 Zhejiang China

**Keywords:** Materials science, Surfaces, interfaces and thin films

## Abstract

Oxygen defects are essential building blocks for designing functional oxides with remarkable properties, ranging from electrical and ionic conductivity to magnetism and ferroelectricity. Oxygen defects, despite being spatially localized, can profoundly alter global properties such as the crystal symmetry and electronic structure, thereby enabling emergent phenomena. In this work, we achieved tunable metal–insulator transitions (MIT) in oxide heterostructures by inducing interfacial oxygen vacancy migration. We chose the non-stoichiometric VO_2-δ_ as a model system due to its near room temperature MIT temperature. We found that depositing a TiO_2_ capping layer on an epitaxial VO_2_ thin film can effectively reduce the resistance of the insulating phase in VO_2_, yielding a significantly reduced R_OFF_/R_ON_ ratio. We systematically studied the TiO_2_/VO_2_ heterostructures by structural and transport measurements, X-ray photoelectron spectroscopy, and ab initio calculations and found that oxygen vacancy migration from TiO_2_ to VO_2_ is responsible for the suppression of the MIT. Our findings underscore the importance of the interfacial oxygen vacancy migration and redistribution in controlling the electronic structure and emergent functionality of the heterostructure, thereby providing a new approach to designing oxide heterostructures for novel ionotronics and neuromorphic-computing devices.

## Introduction

The importance of oxygen point defects in dictating physical properties has been more and more widely recognized by the functional oxide community^1–4^. Oxygen defects, disguised by the name, can be actually used to advantageously enhance functionalities and device performances, ranging from electronic^5–7^, magnetic^8–10^ and multiferroic properties^11^ to energy storage and conversion applications^2,12,13^. Especially, there has been an increasing interest in utilizing oxygen point defects for tailoring electronic structures of oxides, due to the designability and reversibility of this approach^14^. Electrical switching enabled by oxygen defects has been proven promising for applications in neuromorphic computing due to the fact that oxygen defects allow actively tuning electrical conductivity on demand^14,15^. In the pursuit of a defect-tuning functionality in oxides, it was found that the effects of oxygen defects can go beyond what can be explained within the simple rigid band model and band-filling picture. In correlated oxide systems, changes in oxygen content can profoundly alter electronic structure and even trigger a metal–insulator transition (MIT). VO_2-δ_ (*δ* denotes oxygen non-stoichiometry) is such a material system, wherein a small change in composition can lead to a large modulation in correlation effects^16–20^. Near-stoichiometric VO_2_ has 3*d*^1^ electron configuration and shows a MIT with a transition temperature *T*_*c*_ ≈ 340 K, accompanied by a structural transition from low-temperature M1 (monoclinic) phase to high-temperature R (rutile) phase. The MIT transition temperature can be lowered by 60 K using epitaxial strain imposed by substrates^21,22^. Interestingly, the MIT has been shown to be completely suppressed by inducing oxygen vacancy formation, either chemically^17,23^ or electrochemically^16,24^. Oxygen-deficient VO_2-δ_ was shown to remain in the metallic R phase when cooled down to below the MIT transition temperature. This makes oxygen stoichiometry a knob for reversibly controlling the MIT in VO_2_.

Oxygen non-stoichiometries in functional oxide thin films are most commonly manipulated by changing the oxygen electrochemical potential via annealing in a certain oxygen partial pressure (*p*O_2_) or by applying electrical bias^25^. In contrast, a novel strategy based on interfacial oxygen defect migration induced by the mismatch of defect formation energy of two dissimilar oxides, is much less studied. This “oxygen diode” effect essentially utilizes the oxygen chemical potential gradient from the difference in defect formation energy (*E*_*f*_) across oxide interfaces. In order to balance the oxygen chemical potential, a unidirectional flow of oxygen defects can be induced during the fabrication process of oxide heterostructures. Recently, this approach has been successfully applied to tune the charge carrier density of LaNiO_3-δ_ (LNO) by using oxide capping layers with different oxygen vacancy formation energies^26^, and also to transform thin films of La_0.67_Sr_0.33_CoO_3_ (LSCO) from its perovskite form to the brownmillerite form La_0.67_Sr_0.33_CoO_2.5_ by capping thin film with Gd^27^. Since the underlying mechanism of this effect is not material specific, it should be feasible to apply this approach to a broader spectrum of oxide material systems.

In this work, we use a TiO_2_ capping layer to construct “oxygen diodes” of TiO_2_/VO_2_ heterostructures, in order to tune the MIT behavior of VO_2_. Interfacial oxygen vacancy migration from rutile TiO_2_ to VO_2_ is induced by the much higher oxygen vacancy formation energy (*E*_*f*_) of TiO_2_ compared with VO_2,_ as predicted by our ab initio many body diffusion Monte Carlo (DMC) as well as DMC-benchmarked density function theory (DFT + U) based calculations of *E*_*f*_ across this heterostructure. The formation of oxygen vacancies in VO_2_ induced by a TiO_2_ capping layer was also confirmed by an observed *c*-axis lattice expansion using X-ray diffraction (XRD), as well as by electron microscopy and X-ray photoelectron spectroscopy characterization, in agreement with our computational predictions. The MIT behavior in VO_2_ was drastically altered by the incorporation of oxygen vacancies. While a VO_2_ thin film without a TiO_2_ capping layer showed a sharp transition in resistivity vs. temperature, the transition was shown to be suppressed in VO_2_ with TiO_2_ capping layers. Our combined experimental and computational investigation on the TiO_2_/VO_2_ system underscores the effects of oxygen defect redistribution in oxide heterostructures on altering electronic structure and demonstrates how to use the ‘oxygen diode’ effect to control a MIT.

## Methods

### Thin film deposition

VO_2_ and TiO_2_/VO_2_ thin films were grown by using pulsed laser epitaxy (PLE) on TiO_2_ (001) single crystals (CrysTec, Germany). Growth temperature was fixed at 300 °C, and VO_2_ layer was grown in oxygen partial pressure (*p*O_2_) of 15 mTorr, while the TiO_2_ capping layers were grown in three different *p*O_2_, i.e. 10 mTorr, 15 mTorr and 20 mTorr. X-ray diffraction (XRD) was performed on the grown thin films by using a four-circle X-ray diffractometer (Panalytical X’Pert MRD).

### Transport measurement

Electrical contacts to thin film samples were made by using ultrasonic Al wire bonding. A Physical Property Measurement System (PPMS, Quantum Design) was used for measuring resistivity as a function of temperature by performing a warming up and cooling down cycle.

### STEM and EELS

Cross-sectional STEM specimens were prepared to see along the [100]TiO_2_ substrate direction using mechanical thinning and precision polishing followed by ion milling. High-angle annular dark-field (HAADF) imaging and STEM-EELS analysis were carried out in Nion UltraSTEM200 operated at 200 keV. For HAADF imaging, inner detector angle of 65 mrad was used, and the convergence semi-angle for the electron probe was set to 30 mrad.

### X-ray photoelectron spectroscopy (XPS)

XPS measurements were performed on a VO_2_ thin film sample and a VO_2_ thin film with a very thin TiO_2_ capping layer (~ 2 nm). The thin TiO_2_ capping layer was deposited by using the same condition as the LP sample (i.e., 10 mTorr *p*O_2_). The reason for choosing a thin thickness for the TiO_2_ capping layer is due to the shallow probing depth of XPS (estimated ~ 5 nm for V *2p* spectra). XPS spectra of O *1s* and V *2p* were collected by using a system equipped with a monochromated Al Kα X-ray source (*hν* = 1486.7 eV) and a multi-channel detector (Sigma Surface Science, Germany). XPS data collection, as well as XRD and STEM characterizations, were performed at room temperature.

### DFT calculations

Density functional theory (DFT) calculations were performed using the Vienna ab initio simulation package (VASP^28^). Electron exchange correlation was represented by the functional of Perdew, Burke, and Ernzerhof (PBE) of generalized gradient approximation (GGA^29^). The correlation effects were considered by using the DFT + U method^30^ with U values of (U_V_,U_Ti_) = (4.0,5.0) eV as determined by scanning the two-parameter space using many-body quantum Monte Carlo (QMC) calculations (further details available in SI and “QMC calculation” section below). The ion–electron interaction was described with the projector augmented wave (PAW) method^31^. A cutoff energy of 400 eV was used for the plane-wave basis set. The internal coordinates were relaxed until the forces were lower than 0.01 eV/Å. The calculations were performed with spin polarization, and a ferromagnetic (FM) ordering was applied to the V sites.

DFT calculations were performed on 48-atom and 96-atom TiO_2_/VO_2_ supercells. The 48-atom supercell contains four VO_2_ layers and four TiO_2_ layers stacked along the rutile c axis (a = 6.4966 Å, b = 6.4966 Å, c = 11.5892 Å), while the 96-atom supercell contains eight VO_2_ layers and eight TiO_2_ layers (c = 23.1784 Å). Monkhorst–Pack k-point meshes of 6 × 6 × 3 and 6 × 6 × 2 were used for the 48-atom and 96-atom supercells, respectively. The O vacancy formation energy ${E}_{f}^{O}$ was calculated using the equation: ${E}_{f}^{O}={E}^{tot}\left[Vo\right]-{E}^{tot}\left[perf\right]+{\mu }_{O}$, where ${E}^{tot}\left[Vo\right]$ is the total energy of the supercell with one oxygen vacancy, ${E}^{tot}\left[perf\right]$ is the total energy of the pristine supercell, and ${\mu }_{O}$ is the chemical potential of oxygen. We use a definition of ${\mu }_{O}$ common in the literature^32^ as ${\mu }_{O}=\frac{1}{2}$(2 ${E}^{tot}\left[O\right]+ {\varepsilon }_{O2}^{coh}$) where ${E}^{tot}\left[O\right]$ is the energy of an oxygen atom, and ${\varepsilon }_{O2}^{coh}$ is the cohesive energy of oxygen molecule. From experimental data, we determined ${\varepsilon }_{O2}^{coh}$ to be − 5.21 eV^33^.

### QMC calculations

Calculations using the Diffusion Monte Carlo (DMC)^34^ flavor of QMC were performed on 48 atom rutile TiO_2_/VO_2_ interfacial cells containing a neutral oxygen vacancy for all inequivalent sites. The V site magnetic moments were constrained to a ferromagnetic configuration. High quality pseudopotentials^35^ were used to represent the V, Ti, and O species. Fixed node errors were minimized by optimizing LDA + U^36^ parameters for both the V and Ti species separately. All other parameters of the trial wavefunction were optimized via the linear method^37^. All supercell results were averaged over a 2 × 2 × 2 Gamma centered supercell twist grid. The calculations were performed with the QMCPACK simulation code^38^ at the Argonne Leadership Computing Facility and all simulation workflows were driven with the Nexus workflow automation system^39^.

## Results and discussion

**Figure 1:**
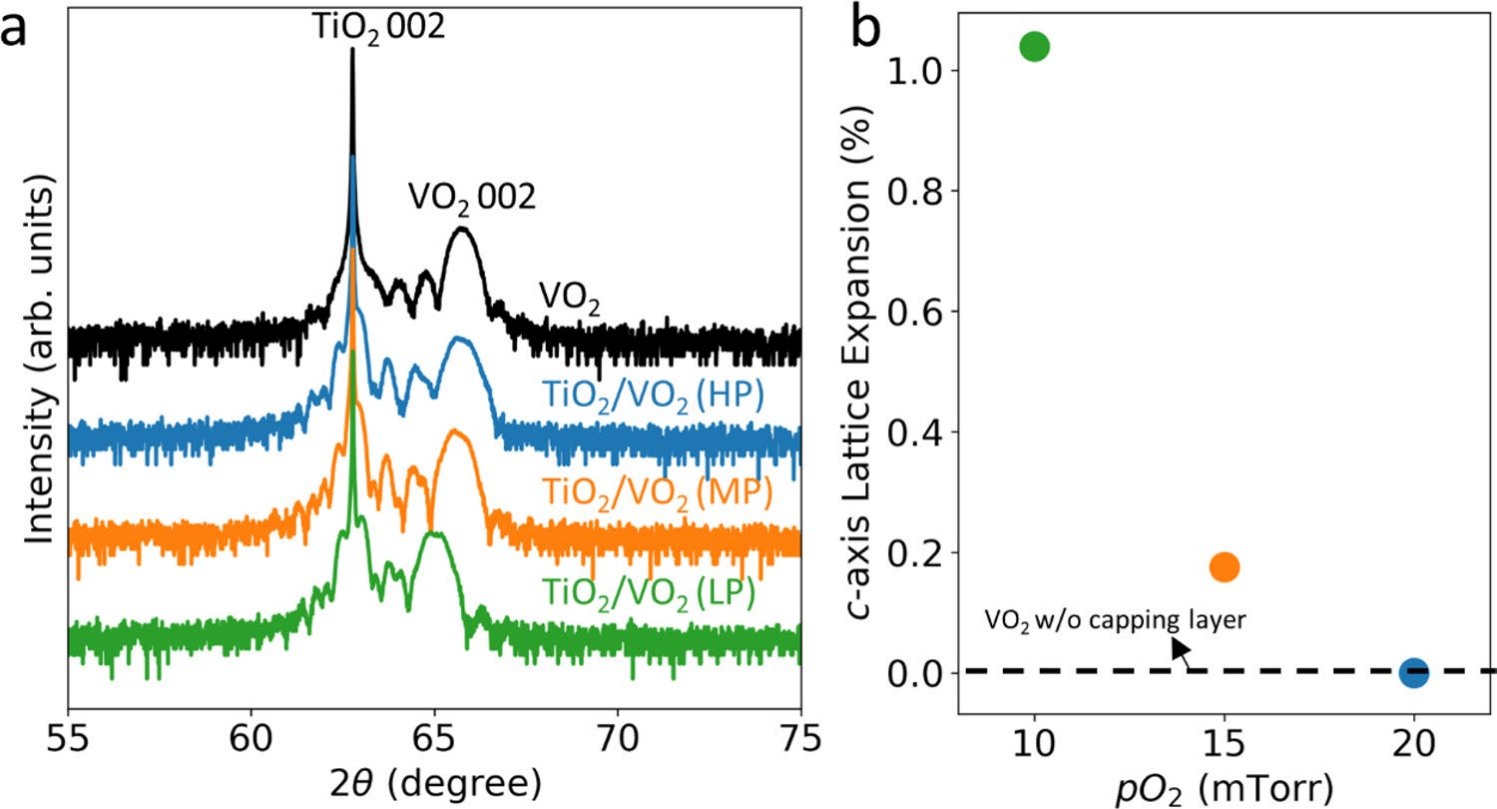
Lattice expansion in VO_2_ induced by interfacial oxygen vacancy migration. **(a)** X-ray diffraction (XRD) results on a VO_2_ thin film (deposited in 15 mTorr *p*O_2_) and VO_2_ thin films (deposited in 15 mTorr *p*O_2_) with TiO_2_ capping layers deposited under different *p*O_2_ of 10, 15, and 20 mTorr (denoted as LP, MP and HP, respectively). **(b)** *c*-axis lattice expansion, compared with a VO_2_ sample without capping layer indicated by the black dashline, as a function of deposition *p*O_2_ for the TiO_2_ capping layer.

VO_2_ thin films and TiO_2_/VO_2_ heterostructures were grown on TiO_2_(001) substrates by pulsed laser epitaxy (PLE) in different oxygen partial pressures (*p*O_2_). The deposition conditions for the VO_2_ layers were fixed to a substrate temperature of 300 °C and 15 mTorr *p*O_2_, while the growth *p*O_2_ of the TiO_2_ capping layers was varied between 10 mTorr, 15 mTorr and 20 mTorr. Both the VO_2_ thin films and the TiO_2_ capping layers were ~ 15 nm thick. We refer to the three TiO_2_/VO_2_ heterostructures grown in different *p*O_2_ as LP, MP and HP (i.e., low, medium and high pressure). The different growth *p*O_2_ of the TiO_2_ capping layer effectively changes the concentration of formed oxygen vacancy in TiO_2_ during deposition. Figure 1 shows XRD scans on VO_2_ and TiO_2_/VO_2_ samples. The clearly resolved thickness fringes indicate the high sample quality. We observed a clear trend of *c*-axis lattice expansion following the direction of decreasing growth *p*O_2_ of TiO_2_ capping layer (i.e. HP < MP < LP). This “chemical expansion”^40^ indicates an increased oxygen vacancy concentration in LP and MP samples compared with bare VO_2_ and HP samples. Therefore, the XRD data is a strong proof of interfacial migration of formed oxygen vacancies in the TiO_2_ capping layer to the VO_2_ layer underneath. A lower growth *p*O_2_ introduces more oxygen vacancies into VO_2_ layer, which results a larger *c*-axis lattice spacing.

**Figure 2:**
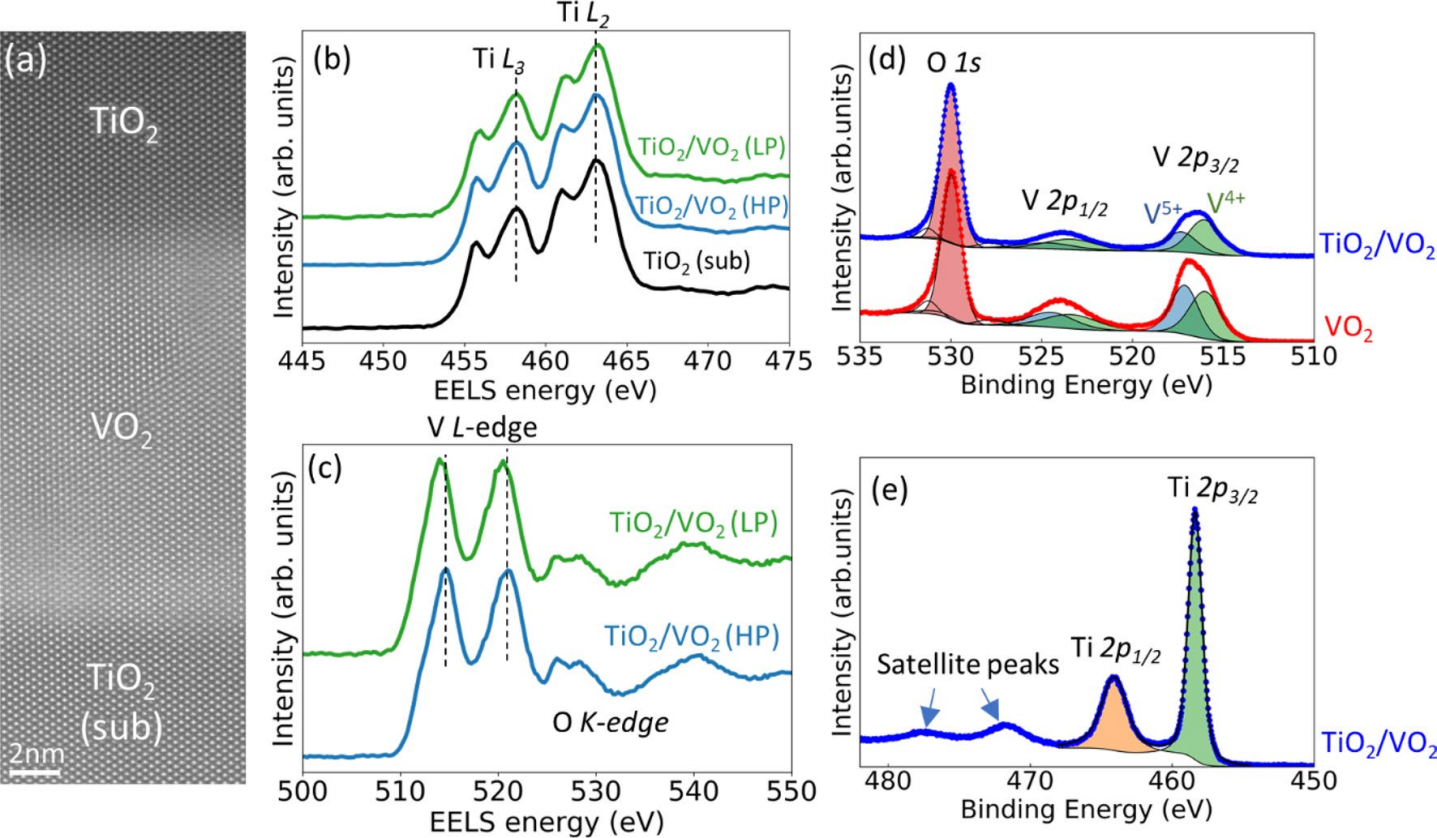
Structure and chemical states of TiO_2_/VO_2_ thin films. **(a)** HAADF-STEM image of TiO_2_/VO_2_ (LP) along the [100] axis of TiO_2_ substrate, which shows high crystal quality without observable extended defects. **(b,c)** Background subtracted EELS spectra of Ti *L*-edge **(b)** and V *L*-edge **(c)**. While there are no appreciable differences between Ti *L*-edge spectra measured on the TiO_2_ capping layers of LP and HP samples, as well as the TiO_2_ substrate, a clear peak shift towards lower energy is observed in V *L*-edge spectrum of LP sample, compared with HP sample. **(d,e)** XPS spectra in V *2p* **(d)** and Ti *2p* **(e)** regions, which provides surface sensitive chemical information. While the Ti *2p* peaks can be fitted with purely 4 + oxidation state, two components representing 4 + and 5 + oxidation states were needed to fit V *2p* peaks. TiO_2_/VO_2_ sample showered higher V^4+^/V^5+^ relative concentration compared with pure VO_2_ sample, which indicates an increased oxygen vacancy concentration induced by the TiO_2_ capping layer.

We also performed detailed structural and chemical analysis to verify the reduction of VO_2_ induced by TiO_2_ capping layers, as shown in Fig. 2. Figure 2a shows a Z-contrast (where Z refers to atomic number) high-angle annular dark-field (HAADF) scanning transmission electron microscopy (STEM) image of a TiO_2_/VO_2_ heterostructure grown on a TiO_2_ substrate. Clearly resolved atom columns and coherent interface again indicate the high sample quality. Figures 2b,c show electron energy loss spectra (EELS) of the Ti *L*_*2,3*_-edge of the TiO_2_ substrate and capping layer, as well as V *L*_*2,3*_-edge of LP and HP samples. The identical line shapes and peak positions in Ti *L*_*2,3*_-edge show the nearly indistinguishable chemical states between TiO_2_ substrate and capping layer. The well-resolved *t*_*2g*_ and *e*_*g*_ peaks indicate that Ti cations in the TiO_2_ capping layer have 4 + oxidation state with no appreciable reduction to 3 + , and do not change valence with decreasing deposition *p*O_2_. In contrast, a clear peak shift towards lower energy was observed in V *L*_*2,3*_-edge spectra comparing LP and HP sample, indicating a lower V oxidation state (high oxygen vacancy concentration) in the LP sample. EELS spectroscopic results on TiO_2_ capping layer and VO_2_ layer evidently reveal that the oxygen vacancies introduced by lowering growth *p*O_2_ of TiO_2_ capping layer migrates to the VO_2_ layer, resulting in the reduced V oxidation state. We believe the atomic-scale oxygen vacancy redistribution in VO_2_ could be affected and non-uniform along the thickness direction due to the vicinity effects associated with the surface and interface. Further investigation to determine both the quantity and spatial nonuniformity of oxygen vacancies would provide useful information to accurately understand the ion transport in VO_2_/TiO_2_ heterostructures.

The unidirectional oxygen vacancy migration from TiO_2_ to VO_2_ was also probed by using X-ray photoelectron spectroscopy (XPS), shown in Fig. 2d,e. Due to the shallow probing depth of XPS (~ 5 nm), we decreased the thickness of the TiO_2_ capping layer down to ~ 2 nm, while interestingly the oxygen diode effect was still apparent. VO_2_ is known to have overoxidized surface layers, therefore a V^5+^ oxidation state was present in the V *2p* spectra^41,42^. Due to the existence of V^5+^ oxidation state, we used the average V oxidation state, represented by the intensity ratio of V^4+^ and V^5+^ (i.e., *I*(V^4+^)/*I*(V^5+^)), as a measure of oxygen vacancy concentration in the VO_2_ layer. As shown in Fig. 2d, the TiO_2_ capping lowered the average oxidation state of V, as indicated by a higher relative intensity of V^4+^ peak (higher *I*(V^4+^)/*I*(V^5+^)). On the other hand, the Ti *2p* spectrum collected on TiO_2_ capping layer (Fig. 2e) can be fitted with only one single component of Ti^4+^, which is consistent with EELS results showing the near absence of oxygen deficiency in TiO_2_ capping layer. We would like to point out that, since the quantitative analysis of oxygen vacancies based on the local EELS data and surface sensitive XPS is challenging, we remain our conclusion be only qualitative.

**Figure 3:**
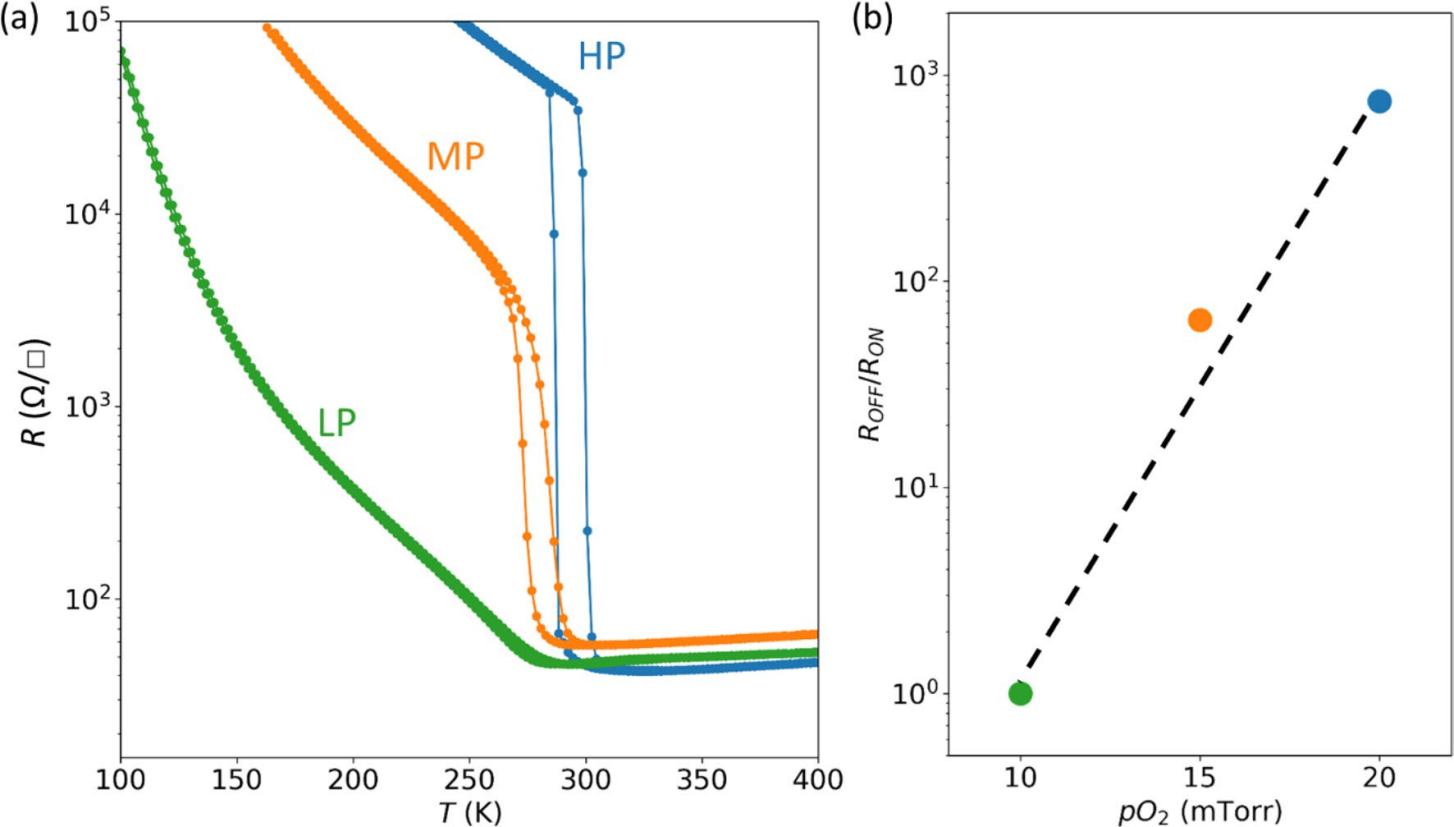
Metal–insulator transition (MIT) tuned by interfacial oxygen vacancy migration. **(a)** Sheet resistance of TiO_2_/VO_2_ LP, MP and HP samples. **(b)** ON/OFF ratio (ratio of resistivity measured during cooling down and warming up cycle at *T*_*c*_, i.e., 278, 287, and 295 K for LP, MP and HP samples, respectively) as a function of deposition *p*O_2_ of the TiO_2_ capping layers. *T*_*c*_ for each sample is defined by taking the average of transition temperatures for cooling down and warming up cycle.

We hypothesize that the increased oxygen deficiency is due to the oxygen diode effect, and results in strongly affecting the MIT of VO_2_, as shown by transport (resistivity ~ temperature plot) data in Fig. 3. We observed a clear MIT in the TiO_2_/VO_2_ HP sample with a sharp change in the resistivity at *T*_*c*_ of ~ 300 K. This is consistent with the XRD data showing that there is no difference between the *c*-axis lattice spacing of HP and bare VO_2_, indicating a nearly identical oxygen stoichiometry between these two samples. Contrarily, the sharp MIT was gradually suppressed with decreasing deposition *p*O_2_*.* While the MP sample showed a much lower ON/OFF ratio (R_OFF_/R_ON_, defined as the ratio of resistance measured during warming up and cooling down exepriments at *T*_*c*_, see Fig. 3b) compared with the HP sample, the resistivity hysteresis was completely suppressed in the LP sample. The observed suppression of the MIT is consistent with a previous report on oxygen deficient VO_2-δ_ thin films^17^. We also note that our finding is consistent with a recent repot on VO_2_/TiO_2_ heterostructures^43^. By utilizing the proposed “oxygen diode effect” we could tune the MIT and thereby change the ON/OFF ratio by three orders of magnitudes. This therefore provides a novel route of controlling oxide electronic properties.

**Figure 4:**
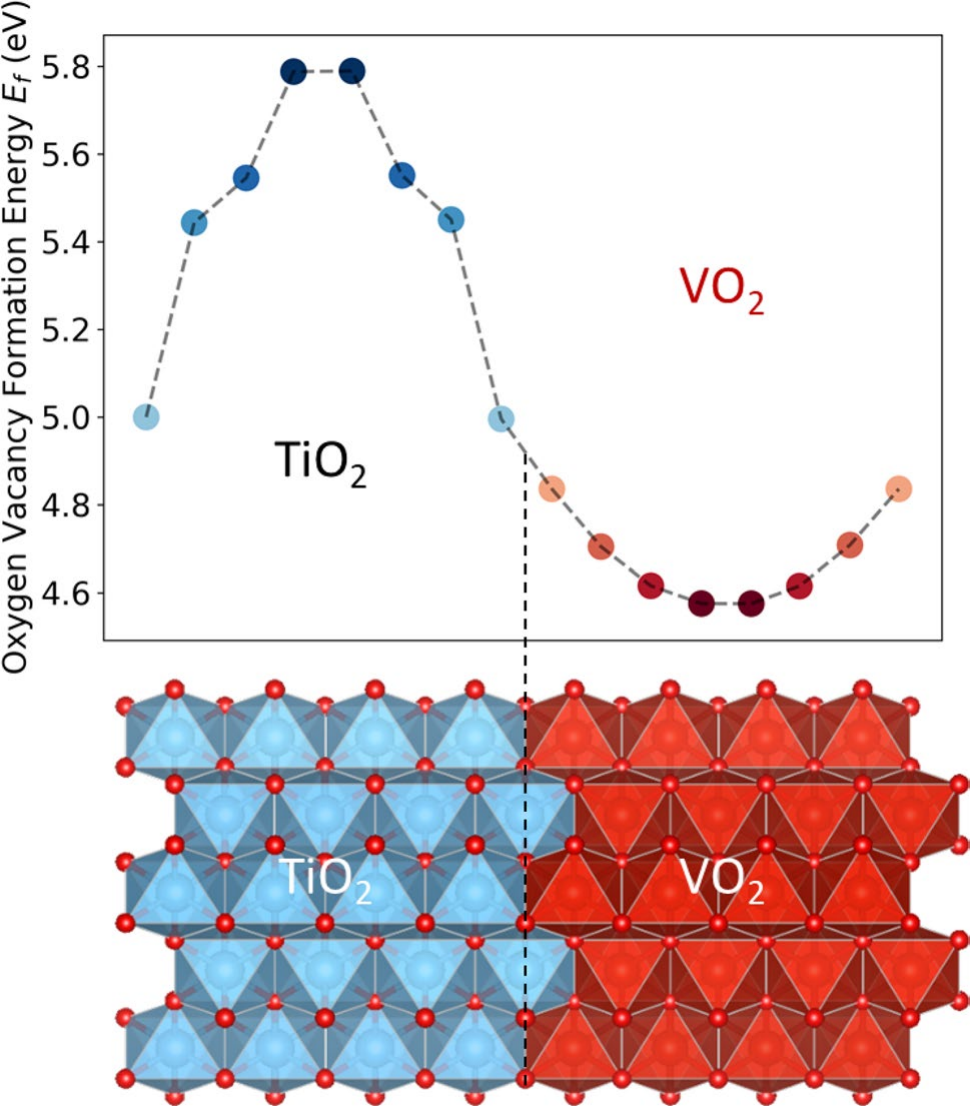
Oxygen vacancy formation energy (*E*_*f*_) landscape. *E*_*f*_ is mapped at different positions of a 96-atom supercell consisted of TiO_2_/VO_2_ bilayer. Oxygen vacancy formation energy was shown significantly lower in the VO_2_ layer compared with the TiO_2_ layer. The dash line indicates the position of TiO_2_/VO_2_ interface.

We further explored the mechanism of the unidirectional oxygen vacancy migration in TiO_2_/VO_2_ by performing ab initio many body diffusion Monte Carlo (DMC) as well as DMC-benchmarked density function theory (DFT + U) calculations. Because of two different 3d metal atoms, we used a hybrid U value of (U_V_,U_Ti_) = (4.0,5.0) eV as benchmarked by surveying the two-dimensional parameter space using total-energies from many-body quantum Monte Carlo (QMC) calculations (further details about the calculations as well as benchmarking can be found in the SI). A 96-atom supercell consisting of TiO_2_/VO_2_ bilayers with an atomically ‘sharp’ interface was used in the DFT calculations to evaluate the (neutral) oxygen vacancy formation energy (*E*_*f*_) in each oxygen atomic layer, as shown in Fig. 4. A gradient of *E*_*f*_ across the TiO_2_/VO_2_ interface was observed, with *E*_*f*_ decreasing from the TiO_2_ layer to the VO_2_ layer. *E*_*f*_ at oxygen layers away from the interfaces approaches the bulk *E*_*f*_ value of TiO_2_ (~ 5.8 eV) and VO_2_ (~ 4.6 eV). In spite of quantitative differences (Fig. S2, discussion in SI), that can be rationalized by comparing the underlying electronic-structure changes (Fig S3), the considerably lower (~ 1 eV) formation energy of oxygen vacancies in VO_2_ compared to TiO_2_ is further supported by benchmark DMC calculations performed in a smaller 48-atom supercell. These DMC calculations provide further support for a solid theoretical justification for the hypothesis that the TiO_2_/VO_2_ heterostructure behaves as an “oxide diode”, similar to the perovskite heterostructures^26^. Allowing effects of cation intermixing to model more realistic interface geometries, at the otherwise ‘sharp’ interface model, does not change this conclusion (Fig. S4). This implies that there is a strong chemical driving force for oxygen vacancy migration from TiO_2_ layer to VO_2_ layer, which is responsible for tuning the oxygen non-stoichiometry, and thereby achieving the suppression of the MIT, that we achieved by using TiO_2_ capping layers grown at different pO_2_ conditions. Even though we did not model the dynamic process of oxygen migration from TiO_2_ to VO_2_, we would like to point out that the growth *p*O_2_ of the TiO_2_ capping layer is the only parameter we altered. Since oxygen vacancies can be readily introduced into grown TiO_2_ by lowering growth *p*O_2_ (See Figure S5 in SI), the only feasible mechanism that responsible for the observed suppressed MIT is the oxygen vacancy mirgration we discussed above.

## Conclusions

In summary, by utilizing the “oxygen diode effect” in TiO_2_/VO_2_ heterostructure we have demonstrated tunability of the MIT and thereby changes in the ON/OFF resistivity ratio by three orders of magnitudes. This, therefore provides a novel route of controlling oxide electronic properties. Electronic-structure calculations as well as multiple characterization methods including XRD, STEM-EELS and XPS were used to prove the unidirectional oxygen vacancy migration across the interface from TiO_2_ to VO_2_. Our results highlight the importance of oxygen defect redistribution by design in oxide heterostructures on determining physical properties and functionalities.

## Supplementary information

Supplementary Information.

## Data Availability

The data that support the findings of this study are available in the supplementary material and from the corresponding author upon reasonable request.

## References

[1] Herklotz, A. *et al.* Strain coupling of oxygen non-stoichiometry in perovskite thin films. *J. Phys. Condens. Matter* **29** (2017). 10.1088/1361-648X/aa949b 29130456

[2] Li Y, Chueh WC (2018). Electrochemical and chemical insertion for energy transformation and switching. Annu. Rev. Mater. Res..

[3] Bishop, S. R., Tuller, H. L., Kuru, Y. & Yildiz, B. Chemical expansion of nonstoichiometric Pr_0.1_Ce_0.9_O_2-δ_: Correlation with defect equilibrium model. *J. Eur. Ceram. Soc.* **31**, 2351–2356 (2011).

[4] Sharma Y (2018). Nanoscale control of oxygen defects and metal-insulator transition in epitaxial vanadium dioxides. ACS Nano.

[5] Jeen H (2013). Reversible redox reactions in an epitaxially stabilized SrCoO_x_ oxygen sponge. Nat. Mater..

[6] Lu N (2017). Electric-field control of tri-state phase transformation with a selective dual-ion switch. Nature.

[7] Meyer, T. L. *et al.* Strain control of oxygen kinetics in the Ruddlesden-Popper oxide La_1.85_Sr_0.15_CuO_4_. *Nat. Commun.* **9**, 92 (2018). 10.1038/s41467-017-02568-z PMC5758782 29311690

[8] Jeen, H. *et al.* Topotactic phase transformation of the brownmillerite SrCoO2.5 to the perovskite SrCoO_3-δ_*Adv. Mater.* **25**, 3651–3656 (2013). 10.1002/adma.201300531 23852832

[9] Walter J (2018). Giant electrostatic modification of magnetism via electrolyte-gate-induced cluster percolation in La_1-x_Sr_x_CoO_3-δ_. Phys. Rev. Mater..

[10] Walter, J. *et al.* Ion-gel-gating-induced oxygen vacancy formation in epitaxial La_0.5_Sr_0.5_ CoO_3-δ_ films from in operando x-ray and neutron scattering. *Phys. Rev. Mater.* **1**, 071403 (2017).

[11] Kalinin, S. V. & Spaldin, N. A. Functional ion defects in transition metal oxides. *Science (80).* **341**, 858–859 (2013). 10.1126/science.1243098 23970692

[12] Petrie JR, Jeen H, Barron SC, Meyer TL, Lee HN (2016). Enhancing perovskite electrocatalysis through strain tuning of the oxygen deficiency. J. Am. Chem. Soc..

[13] Chueh WC, Haile SM (2012). Electrochemistry of mixed oxygen ion and electron conducting electrodes in solid electrolyte cells. Annu. Rev. Chem. Biomol. Eng..

[14] Zhang H-T (2019). Beyond electrostatic modification: Design and discovery of functional oxide phases via ionic-electronic doping. Adv. Phys. X.

[15] Fong DD, Ramanathan S (2017). Preface for special topic: Ionotronics. APL Mater..

[16] Jeong J (2013). Suppression of metal-insulator transition in VO_2_ by electric field-induced oxygen vacancy formation. Science.

[17] Zhang Z (2017). Evolution of metallicity in vanadium dioxide by creation of oxygen vacancies. Phys. Rev. Appl..

[18] Ji H, Wei J, Natelson D (2012). Modulation of the electrical properties of VO_2_ nanobeams using an ionic liquid as a gating medium. Nano Lett..

[19] Brahlek M (2017). Opportunities in vanadium-based strongly correlated electron systems. MRS Commun..

[20] Ganesh p (2020). Doping a bad metal: Origin of suppression of metal-insulator transition in non-stoichiometric VO_2_. Phys. Rev. B.

[21] Quackenbush NF (2013). Nature of the metal insulator transition in ultrathin epitaxial vanadium dioxide. Nano Lett..

[22] Aetukuri NB (2013). Control of the metal-insulator transition in vanadium dioxide by modifying orbital occupancy. Nat. Phys..

[23] Lee S, Meyer TL, Park S, Egami T, Lee HN (2014). Growth control of the oxidation state in vanadium oxide thin films. Appl. Phys. Lett..

[24] Jeong J (2015). Giant reversible, facet-dependent, structural changes in a correlated-electron insulator induced by ionic liquid gating. Proc. Natl. Acad. Sci..

[25] Maier, J. *Physical Chemistry of Ionic Materials: Ions and Electrons in Solids* Vol. 1 (Wiley, 2004).

[26] Guo EJ (2018). Oxygen diode formed in nickelate heterostructures by chemical potential mismatch. Adv. Mater..

[27] Kirby BJ (2018). Ionic tuning of cobaltites at the nanoscale. Phys. Rev. Mater..

[28] Kresse, G. & Furthmüller, J. Efficient iterative schemes for ab initio total-energy calculations using a plane-wave basis set. *Phys. Rev. B Condens. Matter Mater. Phys.* **54**, 11169–11186 (1996). 10.1103/physrevb.54.11169 9984901

[29] Perdew JP, Burke K, Ernzerhof M (1996). Generalized gradient approximation made simple. Phys. Rev. Lett..

[30] Dudarev, S. L., Savrasov, S. Y., Humphreys, C. J. & Sutton, A. P. Electron-energy-loss spectra and the structural stability of nickel oxide: An LSDA+U study. *Phys. Rev. B* **57**, 1505–1509 (1998).

[31] Blöchl PE (1994). Projector augmented-wave method. Phys. Rev. B.

[32] Lindman A, Erhart P, Wahnström G (2015). Implications of the band gap problem on oxidation and hydration in acceptor-doped barium zirconate. Phys. Rev. B.

[33] M.W. Chase, Jr., C.A. Davies, J.R. Downey, Jr., D.J. Frurip, R.A. McDonald, A. N. S. *JANAF Thermochemical Tables*.

[34] Grimm RC, Storer RG (1971). Monte-Carlo solution of Schrödinger’s equation. J. Comput. Phys..

[35] Krogel JT, Santana JA, Reboredo FA (2016). Pseudopotentials for quantum Monte Carlo studies of transition metal oxides. Phys. Rev. B.

[36] Anisimov VI, Zaanen J, Andersen OK (1991). Band theory and Mott insulators: Hubbard U instead of Stoner I. Phys. Rev. B.

[37] Umrigar CJ, Toulouse J, Filippi C, Sorella S, Hennig RG (2007). Alleviation of the fermion-sign problem by optimization of many-body wave functions. Phys. Rev. Lett..

[38] Kent PRC (2020). QMCPACK: Advances in the development, efficiency, and application of auxiliary field and real-space variational and diffusion quantum Monte Carlo. J. Chem. Phys..

[39] Krogel JT (2016). Nexus: A modular workflow management system for quantum simulation codes. Comput. Phys. Commun..

[40] Bishop SR (2014). Chemical expansion: Implications for electrochemical energy storage and conversion devices. Annu. Rev. Mater. Res..

[41] Demeter M, Neumann M, Reichelt W (2000). Mixed-valence vanadium oxides studied by XPS. Surf. Sci..

[42] Quackenbush NF (2015). X-ray spectroscopy of ultra-thin oxide/oxide heteroepitaxial films: A case study of single-nanometer VO_2_/TiO_2_. Materials (Basel)..

[43] Park Y (2020). Directional ionic transport across the oxide interface enables low-temperature epitaxy of rutile TiO_2_. Nat. Commun..

