# Supplementary material for: Metal–insulator transition tuned by oxygen vacancy migration across TiO2/VO2 interface
Source: Sci Rep. 2020 Oct 29;10:18554. doi: 10.1038/s41598-020-75695-1 (PMC7596522; doi:10.1038/s41598-020-75695-1)
Supplement: Supplementary file 1 — Supplementary Information. [file 41598_2020_75695_MOESM1_ESM.docx]

**Supporting Information for** “Metal-Insulator Transition Tuned by Oxygen Vacancy Migration across TiO_2_/VO_2_ interface”

Qiyang Lu^1 # &^, Changhee Sohn^1 #^, Guoxiang Hu^2 #^, Xiang Gao^1^, Matthew F. Chisholm^1^, Ilkka Kylänpää^1,3^, Jaron T. Krogel^1^, Paul R. C. Kent^2,4^, Olle Heinonen^5^, P. Ganesh^2^ and Ho Nyung Lee^1*^

*^1^ Materials Science and Technology Division, Oak Ridge National Laboratory, Oak Ridge, Tennessee 37831, United States*

*^2^ Center for Nanophase Materials and Sciences, Oak Ridge National Laboratory, Oak Ridge, Tennessee 37831, United States*

*^3^ Computational Physics Laboratory, Tampere University, P.O. Box 692, FI-33014 Tampere, Finland*

*^4^ Computational Science and Engineering Division, Oak Ridge National Laboratory, Oak Ridge, Tennessee 37831, United States*

*^5^ Materials Science Division, Argonne National Laboratory, Lemont, Illinois 60439, United States*

*^#^ These authors contributed equally.*

*^*^ hnlee@ornl.gov*

^&^ *Current address:* School of Engineering, Westlake University, Hangzhou, Zhejiang, China 310024

**Supplemental Material**

**Quantum Monte Carlo**

Quantum Monte Carlo (QMC) [1] calculations were performed with the QMCPACK simulation code [2]. All computational workflows were driven by the Nexus workflow automation system [3]. QMC calculations used a 48 atom TiO_2_/VO_2_ cell containing four VO_2_ layers followed by four TiO_2_ layers stacked along the rutile c axis. In all calculations a ferromagnetic (FM) ordering was applied to the vanadium sites. The oxygen vacancy formation energy is dominated by electrostatic effects so choosing FM, AFM, or another ordering of the local moments will not affect the formation energies in a significant way.

Diffusion Monte Carlo (DMC) [4] was used to solve for the ground state of the many body Hamiltonian of each system within the fixed node approximation [5]. The Ti and V ions were represented by Ne core RRKJ pseudopotentials [6] that have been vetted in prior studies [7,8,9,10]. Similarly, a vetted [7,11] He core RRKJ pseudopotential was used for oxygen. The DMC calculations involved 392 electrons for bulk cells and 386 electrons for cells containing neutral oxygen vacancies and the non-local pseudopotentials were handled within the T-move [12] scheme in DMC. A small timestep of 0.005/Ha was used in all DMC calculations, rendering timestep errors negligible on energy differences.

The DMC trial wavefunction used in this study is of the standard Slater-Jastrow form [13,14]. The single particle orbitals defining the Slater determinant, and hence the many body nodal surface, were obtained from DFT [15,16] LDA+U [17] calculations performed with the Quantum
Espresso package [18]. The DFT charge density was converged on a 4x4x4 Gamma centered supercell k-point grid during a self-consistent field calculation. In both preparatory bulk nodal optimization and production oxygen vacancy DMC runs, orbitals were generated via non-self-consistent calculations on a Gamma centered 2x2x2 supercell k-point grid.

In the DFT calculations, a plane wave energy cutoff of 350 Ry was necessary to converge the total energy due the high quality but hard pseudopotentials. Such a large plane wave energy cutoff translates into large memory demands for the real space orbitals represented as B-splines for QMC. In order to reduce memory costs to enable calculations on Mira at the Argonne Leadership Computing Facility, a hybrid representation [19] was used for the orbitals.

The trial Jastrow factor consisted of inhomogeneous one- and homogeneous two-body terms, each represented by sums of 1D B-spline based correlation functions in electron-ion or electron-electron pair distances. The trial Jastrow factor was optimized using the linear method [20].

The many body nodal surface of the trial wavefunction was optimized by scanning over a range of candidate onsite U values for the Ti and V species separately. U values in the range of 1 to 5 eV were considered. The trial Jastrow factor was optimized at a single U value pair, (U_V_,U_Ti_) = (2.0,3.0), and held fixed across the other points to minimize pseudopotential localization error in the relative energies. Optimal U values were selected by first performing LDA+U calculations at each Ti/V onsite U pair followed by fixed node DMC calculations. Since DMC is a variational method, the U values providing the lowest DMC energy correspond to the best wavefunction in the search space. The resulting U_V_, U_Ti_ energy surface is shown in Figure S1, where DMC energies calculated at the points shown by the black dots. Points missing on the regular 1 eV spaced grid correspond to runs where the DFT LDA+U calculations suffered convergence problems in the Quantum Espresso code. Data in between the sampled points was estimated using a 2D cubic spline interpolant. The resulting fixed node energy surface shows that wavefunctions resulting from vanadium onsite U values lower than 3.5 eV are sub-optimal. This agrees with expectations based on our prior studies of bulk VO_2_ [10] where a similar nodal optimization procedure found that a U value of 3.5 eV was optimal, while U values from 3.5 to 5 eV were similar in energy. In this region (U_V_>3.5 eV) the effect of the Ti U value is more clearly seen, leading to multiple nearby minima, with U pairs (U_V_,U_Ti_) = (4.0,5.0), (5.0,4.0) and (5.0,1.0) eV leading to wavefunctions of comparable quality. The differences among these wavefunctions represent variational freedom in the magnetic *d*-manifold of the transition metal species. Given this, and the explicit energetic similarity among the available minima, we are free to select among them without significantly affecting the vacancy formation energies. Indeed, with the optimal U pair, the vacancy formation energy using PBE+U and LDA+U using the PAW pseudo-potentials showed similar relative profile, with an overall constant shift between the two curves (not shown). For sake of consistency, we only show result for structures obtained by all atomic relaxations using the PBE functional in this manuscript.

DMC calculations were performed for all DFT relaxed vacancy structures in the 48 atom cell using selected optimal U values of (U_V_,U_Ti_) = (4.0,5.0) eV. The eight vacancy structures considered are symmetric about either the TiO_2_ or VO_2_ mid-planes, resulting in four pairs of structures identical under inversion. In all cases, only the neutral oxygen vacancy was considered. For one pair of equivalent sites, the LDA+U precursor calculations converged to different minima during the self-consistent calculation. The DMC variational principle was subsequently used to select the best wavefunction, i.e. the one resulting in a lower energy. For all other pairs, the DMC energies agreed statistically. Oxygen vacancy formation energies were calculated using the lowest DMC energies for each pair, along with energies computed for the non-defective interface and a bare oxygen dimer to set the oxygen chemical potential. In the non-defective interface and oxygen vacancy cells, the electronic charge and spin densities were collected on 44x44x72 histogram grids for subsequent electrostatic analysis.

The DMC oxygen vacancy formation energies are shown in Figure S2 along with PBE+U results. The two theories are in agreement regarding the most important qualitative feature of the energy profile, namely that oxygen vacancies preferentially reside in the VO_2_ region. Diffusion Monte Carlo finds oxygen vacancy formation energies about 0.3 eV lower in the TiO_2_ region and 0.6 eV lower in the VO_2_ region than PBE+U. More interestingly, DMC and DFT reveal differing trends in the formation energy as the TiO_2_/VO_2_ interface is traversed. While both theories agree, even quantitatively, that the formation energy decreases as the vacancy crosses from the TiO_2_ region into the first interfacial layer, PBE+U finds a continued decrease as VO_2_ is approached while DMC reveals a distinct barrier.

To get insights into the nature and significance of this difference, we inspected the electronic charge density difference between the defective and pristine structure at both PBE+U and QMC levels of theory, for the different positions of the oxygen vacancy in our 48-atom supercell, as shown in Fig. S3. In general, for all positions of the oxygen vacancies, the density differences seem to be similar between the two levels of theory and converges quickly in the TiO_2_ side of the interface than in the VO_2_ side of the supercell. This is possibly because of the poor screening in the VO_2_ side of the in our small supercell. In particular when the vacancy is closest to the interface, in the VO_2_ side, screening appears to be worst as seen by the anomalously large charge-density differences between the two levels of theory, which doesn’t converge within the VO_2_ slab. This poor screening due to the small VO_2_ size of the slab in our 48-atom supercell could possibly explain the observed quantitative difference in the relative E_f_ between the two oxides as seen in Fig. S4 and discussed above. This finite-size effect should be much smaller in our larger 96-atom supercell calculations.

**TiO_2_ thin films grown under low-pressure condition**

We have grown a TiO_2_ thin film sample under 10 mTorr *p*O_2_, which is the same condition used for the LP sample in the manuscript. XRD shown in Figure S5 revealed that the grown TiO_2_ thin film has larger lattice parameter compared with the TiO_2_ substrate, which indicates the existence of oxygen vacancies inside the TiO_2_ thin film grown under this low *p*O_2_ condition,

**Figure S1. Fixed node DMC energy vs. onsite V and Ti U values.** DMC energies shown are total energies for a 48 atom non-defective TiO_2_/VO_2_ cell relative to the lowest total energy found (-1556.298(6) Ha).


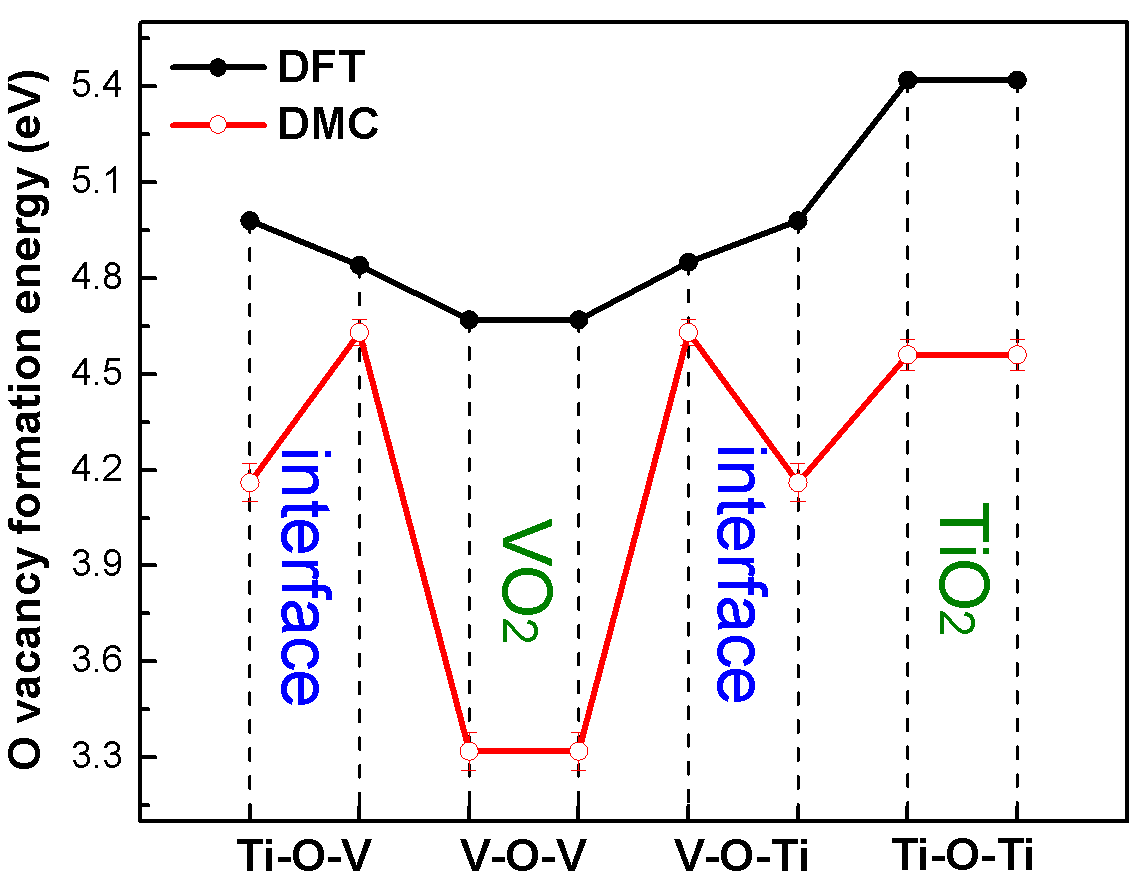


**Figure S2. Fixed node DMC and PBE+U oxygen vacancy formation energies in a 48 atom TiO_2_/VO_2_ cell.** The x-axis of the figure corresponds to the rutile c-axis with formation energies ordered by layer in the cell.


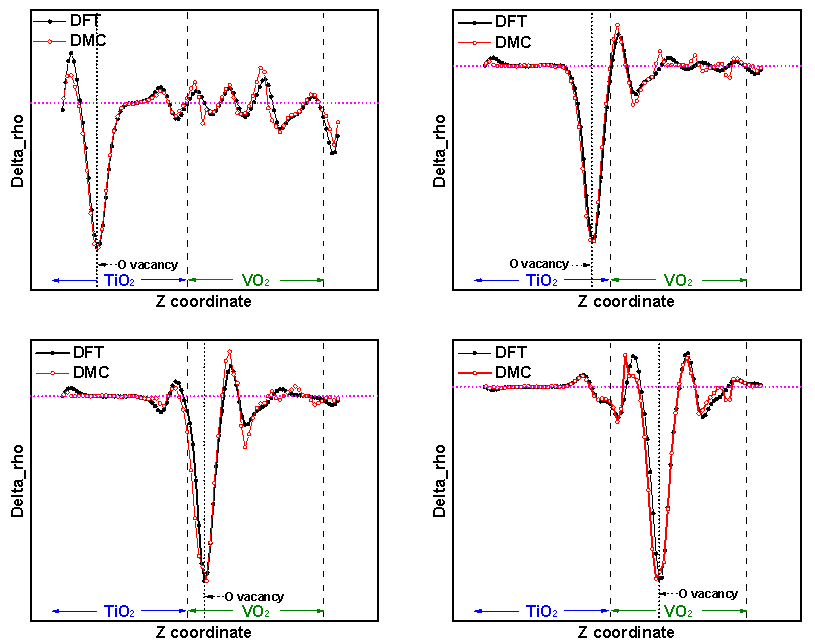


**Figure S3. DMC and PBE+U plane-averaged charge density difference plots for the 48-atom TiO_2_/VO_2_ supercell with O vacancies at different positions.** The charge density was referenced to the pristine 48-atom TiO_2_/VO_2_ supercell.


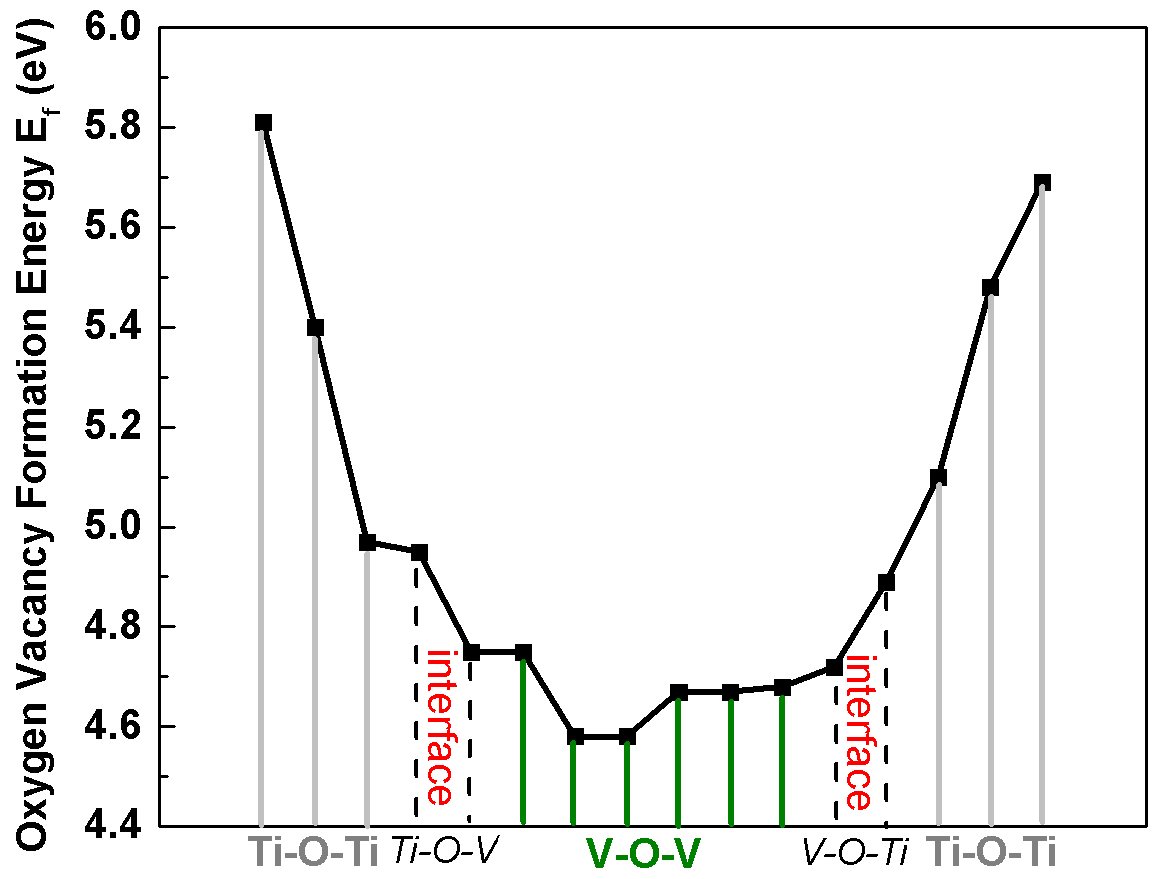


**Figure S4. Oxygen vacancy formation energy *E_f_* for a 96-atom TiO_2_/VO_2_ supercell with the intermixed interface.** Oxygen vacancy formation energy was still significantly lower in the VO_2_ layer compared with the TiO_2_ layer.


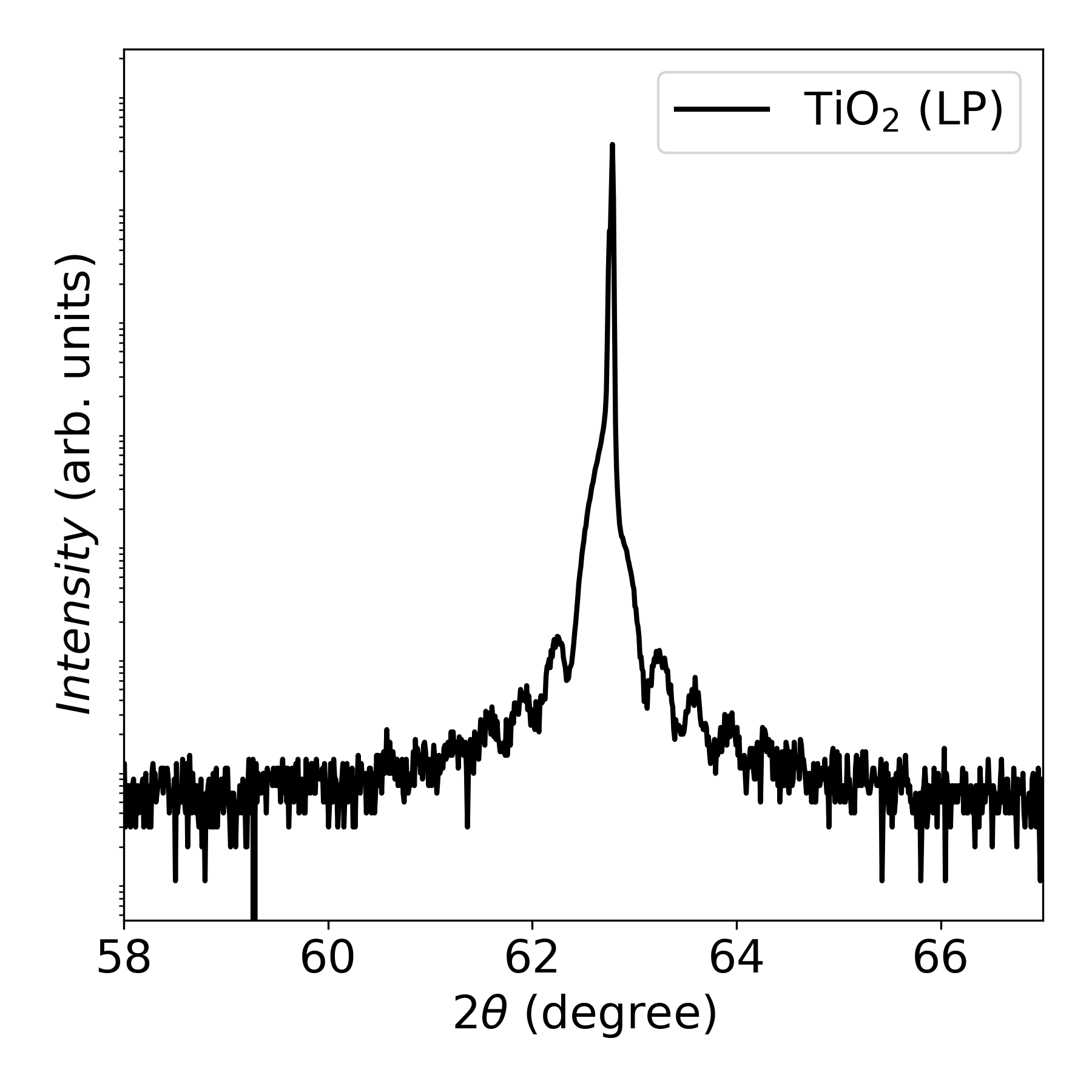


**Figure S5 XRD scan around TiO2 002 peak of a TiO2 (LP) sample grown on TiO2 substrate.** The thin film peak shows a higher shoulder on the left side of the substrate peak, which indicates the lattice expansion of TiO2 thin film compared with the single crystal substrate.

**References**

[1] W. M. C. Foulkes, L. Mitas, R. J. Needs, and G. Rajagopal, *Rev. Mod. Phys.* **2001**, *73*, 33.

[2] J. Kim, A. D. Baczewski, T. D. Beaudet, A. Benali, M. C. Bennett, M. A. Berrill, N. S. Blunt, E. J. Landinez Borda, M. Casula, D. M. Ceperley, S. Chiesa, B. K. Clark, R. C. Clay III, K. T. Delaney, M. Dewing, K. P. Esler, H. Hao, O. Heinonen, P. R. C. Kent, J. T. Krogel, I. Kylänpää, Y. W. Li, M. G. Lopez, Y. Luo, F. D. Malone, R. M. Martin, A. Mathuriya, J. McMinis^9^, C. A. Melton, L. Mitas, M. A. Morales, E. Neuscamman, W. D. Parker, S. D. Pineda Flores, N. A. Romero, B. M. Rubenstein, J. A. R. Shea, H. Shin, L. Shulenburger, A. F. Tillack, J. P. Townsend, N. M. Tubman, B. Van Der Goetz, J. E. Vincent, D. C. Yang, Y. Yang, S. Zhang and L. Zhao, *Journal of Physics: Condensed Matter* **2018**, *30*, 195901.

[3] J. T. Krogel, *Computer Physics Communications* **2016**, *198*, 154.

[4] R. Grimm and R. Storer, *J. Comput. Phys.* **1971**, *7*, 134.

[5] J. B. Anderson, *J. Chem. Phys.* **1975,** *63*, 1499.

[6] A. M. Rappe, K. M. Rabe, E. Kaxiras, and J. D. Joannopoulos, *Phys. Rev. B* **1990,** *41*, 1227.

[7] J. T. Krogel, J. A. Santana, and F. A. Reboredo, *Phys. Rev. B* **2016**, *93*, 075143.

[8] Y. Luo, A. Benali, L. Shulenburger, J. T. Krogel, O. Heinonen, P. R. C. Kent, *New Journal of Physics* **2016,** *18*, 113049.

[9] A. Benali, L. Shulenburger, J. T. Krogel, X. Zhong, P. R. C. Kent, O. Heinonen, *Phys. Chem. Chem. Phys.* **2016**, *18*, 18323.

[10] I. Kylänpää, J. Balachandran, P. Ganesh, O. Heinonen, P. R. C. Kent, J. T. Krogel, *Phys. Rev. Materials* **2017**, *1*, 065408.

[11] J. A. Santana, J. T. Krogel, J. Kim, P. R. C. Kent, F. A. Reboredo, *The Journal of Chemical Physics* **2015**, *142*, 164705.

[12] M. Casula, *Physical Review B* **2006**, *74*, 161102.

[13] J. C. Slater, *Phys. Rev.* **1929**, *34*, 1293.

[14] R. Jastrow, *Phys. Rev.* **1955**, *98*, 1479.

[15] P. Hohenberg and W. Kohn, *Phys. Rev.* **1964**, *136*, B864.

[16] W. Kohn and L. Sham, *Phys. Rev.* **1965**, *140*, A1133.

[17] V. Anisimov, J. Zaanen, and O. Andersen, *Physical Review B* **1991**, *44*, 943.

[18] P. Giannozzi, S. Baroni, N. Bonini, M. Calandra, R. Car, C. Cavazzoni, D. Ceresoli, G. L. Chiarotti, M. Cococcioni, I. Dabo, A. D. Corso, S. de Gironcoli, S. Fabris, G. Fratesi, R. Gebauer, U. Gerstmann, C. Gougoussis, A. Kokalj, M. Lazzeri, L. Martin-Samos, N. Marzari, F. Mauri, R. Mazzarello, S. Paolini, A. Pasquarello, L. Paulatto, C. Sbraccia, S. Scandolo, G. Sclauzero, A. P. Seitsonen, A. Smogunov, P. Umari, R. M. Wentzcovitch, *J. Phys. Condens. Matter* **2009**, *21*, 395502.

[19] Y. Luo, K. P. Esler, P. R. C. Kent, L. Shulenburger, *J. Chem. Phys.* **2018**, *149*, 084107.

[20] C. J. Umrigar, J. Toulouse, C. Filippi, S. Sorella, R. G. Hennig, *Phys. Rev. Lett.* **2007**, *98*, 110201.
